# Meteorological Observations Taken

**Published:** 1859-07

**Authors:** J. G. Westmoreland

**Affiliations:** The Smithsoman Institution, June, 1859, at Atlanta, Ga.


					﻿! « i
®	>	fcj >: W	Ji . . . -pqis;
T-i	>2i!2i	co	”02	03	“'^'^ SqoQ
’O i
ps '	--------^ - - - .............. -_______________________________
it'	!25!2;	02	MM tzi’^ai
S 11
rO H	o
« it	s
p^ ‘^®®OC>OCOi0OOii3O OiO oooooc>io®cnoo»oooo "S
*2.^ i o=>	'"' ’~"^	®
c5 J ! M	~ ~	-----------------------
. O	1 o	U
co j P	o
®	o	o'	®®2®®^'-’®‘^’^O‘0‘C)i0C>Oi0OOi0OiCO>0'»OOOOO	3
t-p'-'	,—1,—(	„	I-,^
g §	___________,	_	____________________ -
‘"g. ''	.	’3
o S I i
S *5 I'	•	® ®	® ®o lO lO IC IC O O O O »O O »O 10 O O lO o o o o o £
rti-lrlrtn-lrt	CH
I !i	'3
gj j! ..______________________ «
i! 5	®
co	ij----5—--------------------------------------------------------------  3
■'	.	s	"
’^••s	ii	e-i	=»	S
^t-2 '; s	«
O .•	^SSf®7t!?’?°S52?SSJ;3?®®®'^'®®®®MQ0O50O'c»<(NeDC>0COO	2
Q . i in	^°tIt-^*>’t'"l'~GOCOMQOOOl'-QOC0 1— r-OOOO^l'-OOl'-OOXOOOOOQOOOO 5
e	i	2	<M	i
?*	I	t3
r5S S J	co o 1- co co co o o <£> co co t- t- S S S S t* r- I- I'- t-
s	g	I______>'__________________________________________________________     «
C	>5	i	*5	®2'®®‘^!£?*^t'®'^®’^'^‘-'5C<|lOOiOOOOXOO<MOOOtOi(5lOC>
Oq^	«	— '-Hr-HOMCJC'IOI — 05C3C>O05WC5C>i-i>-iIM<MC0C'I01
C	’	p<	2 5®®'®®®'^'^®®-cj6d6c3sdiT?no;ooocooc6
P	—	COCOCOCOCOCOCOPOCOOOCOCOCOCOCOC^(MCOCOC<l<>i;^ICOCOCiOCOCOCOCOCO
W	_____________________ __________
i	C2	OIWMi-<C<IOO.'-l05<>J-MC<]SqeOCOtNOcnOr-lCi-IC53.C^C4(MOO'cJfCC(M
e*	j5	o®oooooc>os’c>ooooc>c50c)0(OC5C5000oooc!
I	cococococococococococococococcccc<icococococ-icocococococococo
8 I rt J=^___________________________________________________________________
P	2 si «30 0, OC^OO‘OlOOlOV5iOt'.(Nb-OiOCOOOu'?OqOC<IOiaOO»0
R	P	’-;C'i’-JO'-^C'4C<l<>J>-H,-’c-lC<IC;|C^'M005030C00005i-lr-<<MCCOC.OCl
P	<	SSSSS2S2S2‘^®®p'o®'^'®> POO’O’05 0000000
|j»	co co co co co co KI co co cococococococo<N<Mcocooicioicocococoeococo
5	Uav of Mo. I '-' Ot «) T*1 lO co I-^ 03 05 O i-CC<ICO'<^<10«5t^OOC50i-l C^CO^ 10 O t- 00 05 o
'	'-ii-’i-it-ir-Hi-li-(r-tr-1i-lC4<MC<,CMMC-, dC^OKNCO
				

